# The Effects of 1 mA tACS and tRNS on Children/Adolescents and Adults: Investigating Age and Sensitivity to Sham Stimulation

**DOI:** 10.1155/2020/8896423

**Published:** 2020-08-13

**Authors:** Maike Splittgerber, Jan Hendrik Suwelack, Navah Ester Kadish, Vera Moliadze

**Affiliations:** Institute of Medical Psychology and Medical Sociology, University Medical Center Schleswig Holstein, Kiel University, Kiel, Germany

## Abstract

The aim of this study was to investigate the effect of transcranial random noise (tRNS) and transcranial alternating current (tACS) stimulation on motor cortex excitability in healthy children and adolescents. Additionally, based on our recent results on the individual response to sham in adults, we explored this effect in the pediatric population. We included 15 children and adolescents (10–16 years) and 28 adults (20–30 years). Participants were stimulated four times with 20 Hz and 140 Hz tACS, tRNS, and sham stimulation (1 mA) for 10 minutes over the left M1_HAND_. Single-pulse MEPs (motor evoked potential), short-interval intracortical inhibition, and facilitation were measured by TMS before and after stimulation (baseline, 0, 30, 60 minutes). We also investigated aspects of tolerability. According to the individual MEPs response immediately after sham stimulation compared to baseline (Wilcoxon signed-rank test), subjects were regarded as responders or nonresponders to sham. We did not find a significant age effect. Regardless of age, 140 Hz tACS led to increased excitability. Incidence and intensity of side effects did not differ between age groups or type of stimulation. Analyses on responders and nonresponders to sham stimulation showed effects of 140 Hz, 20 Hz tACS, and tRNS on single-pulse MEPs only for nonresponders. In this study, children and adolescents responded to 1 mA tRNS and tACS comparably to adults regarding the modulation of motor cortex excitability. This study contributes to the findings that noninvasive brain stimulation is well tolerated in children and adolescents including tACS, which has not been studied before. Finally, our study supports a modulating role of sensitivity to sham stimulation on responsiveness to a broader stimulation and age range.

## 1. Introduction

Noninvasive transcranial brain stimulation (NTBS) may modulate cortical excitability, outlasting the period of NTBS itself from several minutes to more than one hour [1]. Transcranial magnetic stimulation (TMS) and transcranial direct current stimulation (tDCS) are the most commonly used methods of NTBS [1]. Transcranial alternating current stimulation (tACS) is an increasingly popular NTBS technique [2], with the advantage of enabling manipulation and entrainment of intrinsic oscillations through the injection of sinusoidal currents [3–5]. The transcranial random noise stimulation (tRNS) paradigm was developed with a potential to desynchronize normal and pathological cortical rhythms. The frequency band of tRNS can encompass a full range (typically from 0.1 to 640 Hz) or can be delivered at low (0.1–100 Hz)- or high-frequency (101–640 Hz). The concept of tRNS is to enhance the stochastic dynamics of neurons and thus facilitate the neural processing and the related behavior [6, 7] for review see [8].

Until recently, NTBS has been mainly investigated in adults, while studies in children and adolescents are still limited, focusing on TMS and tDCS [9]. Yet, effects in this group are of interest, as they might have accelerated neural plasticity compared to adults after brain stimulation [10]. Therefore, NTBS is expected to have even greater potential to regulate and enhance plasticity in the pediatric population. Indeed, rather than considering it as a small adult brain, a child's brain should be considered as a unique physiological entity [11, 12]. At the same time, extreme caution is needed while dealing with a developing brain, mainly because of a lack of translational studies from adults to children [13].

In the pediatric population, tRNS has rarely been applied [14] and tACS has not yet been studied. In fact, investigating oscillation-specific effects in children is of special interest, e.g., developing the basis for potential treatment options (tRNS/tACS) with a promising safety profile in a vulnerable young population. Therefore, we investigated tRNS and tACS with frequencies both within and outside the conventional electroencephalography (EEG) frequency range (20 Hz and 140 Hz) in children and adolescents. In adults, stimulation in the beta frequency range (~13–30 Hz) have been studied extensively; using EEG, they are linked to a variety of cognitive and sensorimotor processes [15]. For example, Pogosyan and colleagues used a stimulation frequency of 20 Hz, a prominent beta band oscillatory frequency found in the motor system, to study the effect of tACS on movement speed [16]. The results show that, while reaction times were not affected, the subject's voluntary movements were decreased in velocity. Additionally, more recent studies show that tACS in the ripple range (especially 140 Hz) can modulate cortical excitability [17, 18]. tRNS in healthy adults can modulate cortical excitability and improve high-level cognitive functions [7, 19–21].

In this study, we aim to understand the factors determining the efficacy of NTBS and individual differences in response in relation to age. A classical experimental design was chosen in order to compare the results of the current study with previous results obtained in healthy adults [7, 18, 22, 23]. Specifically, the aim of the current study was:.

1. To provide an exploratory investigation of tRNS and 20 Hz as well as 140 Hz tACS in children and adolescents comparing them to adults. The exploratory nature of our analysis is based on the following assumptions: on the one hand, children generally show increased plasticity relative to adults and are thus expected to respond more favourably to noninvasive brain stimulation. However, on the other hand, 1 mA anodal tDCS shows the same excitatory effect both in children and adults [24]. Based on the excitatory nature of 1 mA 140 Hz tACS and tRNS [7, 18, 23], one could therefore expect excitatory effects for these stimulation types for all ages. Additionally, in the case of tRNS and tACS, not only intensity but also frequency plays a role in how it affects the brain. Therefore, we cannot predict the influence of frequency in the developing brain.

Regarding 20 Hz, previous results are heterogeneous with some studies showing an inhibitory effect in adults (for review, see [25]); therefore, this too is treated as an exploratory hypothesis.

(2) In the light of the lack of respective research in pediatrics, we also investigated aspects of tolerability for tACS and tRNS.
(3) In our recent study [22], we explored whether neurophysiological response to sham over the motor cortex could influence response to active stimulation. Response to sham was evaluated based on changes in MEPs immediately after sham stimulation compared to baseline MEPs with a Wilcoxon signed-rank test. We found that subjects who responded to sham stimulation turned out to be nonresponders to verum stimulation when applying tRNS and 140 Hz tACS, while nonresponders to sham showed the expected effects to verum stimulation. Based on this role of the individual response to sham in adults, we explored this effect in the pediatric population. We were therefore interested to see whether sensitivity to sham affects response to verum stimulation and whether a possible effect might be more predictive of response than age.

## 2. Materials and Methods

The study was part of a project investigating different factors which influence variability of tACS and tRNS [22]. Experimental procedures were approved by the local ethics committee of the Kiel University, Kiel, Germany. All participants and their parents were instructed about the study, and written informed consent according to the Declaration of Helsinki on biomedical research involving human subjects was obtained.

### 2.1. Subjects

We included 15 healthy children and adolescents (8 males) aged 10–16 years (M 13.3; SD 2.1) and 28 healthy young adults (19 males) aged 20–30 years (M 24.4; SD 2.5; for details see Table 1). The adult sample in this study has been included in our previous study [22]. All participants were right-handed according to the Edinburgh Handedness Inventory [26]. Exclusion criteria were pregnancy, history of migraine, unexplained loss of consciousness, or brain related injury, IQ <90, history or family history of epileptic seizures, history of other neurological, psychiatric or chronical internistic disorders, intake of central nervous system-effective medication, brain- or cardiac-pacemakers, or not removable metal head implants.

### 2.2. Stimulation Techniques

tACS/tRNS was delivered by a DC stimulator (NeuroConn GmbH, Ilmenau, Germany) through a pair of saline-soaked rectangular sponge electrodes (5 × 7 cm). The motor cortex electrode was fixed over the area representing the right first dorsal interosseus (FDI) muscle as identified by TMS. The other electrode was fixed over the contralateral supraorbital area. This electrode setup has been shown to be the optimal combination to enhance excitability of the M1 [27]. The electrodes were held in place by rubber bands. Stimulation was applied at 20, 140 Hz, tRNS, and sham for 10 minutes. Peak-to-peak current intensity was 1 mA (between -0.5 mA and 0.5 mA). Ramping at the beginning and the end of the stimulation was 5 s in all stimulation conditions. In the sham condition, 30 s of tACS was applied.

The waveform of the 20 Hz and 140 Hz stimulation was sinusoidal (no DC offset, no phase shift). For whole spectrum tRNS in the stimulation mode “noise,” there was a random level of current generated for every sample (sampling rate 1280 sps). The random numbers were normally distributed; the probability density function followed a bell-shaped curve. In the frequency spectrum, all coefficients had a similar size (“white noise”). The noise signal contained all frequencies up to half of the sampling rate, i.e., a maximum of 640 Hz. Due to the statistical characteristics, the signal had no DC offset.

### 2.3. Monitoring of Motor Cortical Excitability

Stimulus intensities of TMS were measured as percentage of maximal stimulator output (MSO %) and determined at the beginning of each experiment. To detect changes in cortical excitability, MEPs of the right FDI were recorded following a single-pulse TMS of its representation area on M1. A Magstim 200 magnetic stimulator (Magstim Company, Whiteland, Wales, UK) with a figure-of-eight standard double magnetic coil (diameter of one winding 70 mm; peak magnetic field 2.2 T; average inductance 16.35 *μ*H) was used. A surface electromyogram (EMG) was recorded from the right FDI through a pair of Ag-AgCl surface electrodes in a belly tendon montage (Nihon Kohden Europe, Rosbach, Germany). The amplified raw-data was band-pass filtered (2 Hz–2 kHz; sampling rate, 5 kHz) and digitized with a micro 1401 AD converter (Cambridge Electronic Design, Cambridge, UK) controlled by Signal Software (Cambridge electronic Design, version2.13). For offline analysis, data was stored on a computer. Complete relaxation was controlled through visual feedback of EMG activity; in case of tension, the subject was reminded to relax. The eight-curved coil was held tangentially to the skull at 45° from the sagittal-line, which results in a posterior to anterior direction of current flow in the brain. The optimum position was defined as the site where TMS resulted consistently in the largest and most stable MEP in the resting muscle. This spot was marked with a skin marker pencil to ensure that the coil was held in the correct position throughout the experiment.

#### 2.3.1. Motor Threshold Determination

The resting motor threshold (RMT) was determined as the minimum stimulator output needed to produce a response of at least 50 *μ*V in the relaxed FDI in at least 3 of 6 consecutive trials. The active motor threshold (AMT) was defined as the lowest stimulus intensity at which 5 out of 10 consecutive stimuli elicited reliable MEPs (above 200 *μ*V in amplitude) during isometric contraction of the contralateral FDI muscle in at least 3 of 6 recordings [28, 29].

#### 2.3.2. Single-Pulse MEPs (SI 1 mV)

The intensity required to evoke a MEP of ~1 mV peak-to-peak amplitude (SI 1 mV) and a baseline of TMS-evoked MEPs (20 stimuli) were recorded at 0.25 Hz.

#### 2.3.3. Intracortical Inhibition and Facilitation

Changes in intracortical excitability were monitored using short-interval intracortical inhibition (SICI) and intracortical facilitation (ICF). A conditioning stimulus (CS, first pulse) was set to 80% of the AMT, while the test pulse (TS, second stimulus) was set to the SI 1 mV threshold. The conditioning stimulus inhibits the MEP amplitude elicited by the test stimulus at short interval (1–5 ms), whereas it facilitates it at longer interval (6–20 ms) [30, 31]. In the present study, we measured SICI at 2 ms ISI, because inhibition was reported to be maximal and expressed without contamination by short-interval intracortical facilitation (SICF), ICF, or any refractoriness of neural elements at this interval [32–34]. For ICF, we chose an ISI of 12 ms, because we expected a maximal increase in MEP amplitude at the median ISI (6–20 ms) known to induce MEP facilitation [35]. We recorded 15 MEPs evoked by the TS and 15 MEPs evoked by the paired pulses (CS + TS) for SICI and ICF, separately.

### 2.4. Experimental Design and Procedure

**Figure 1:**
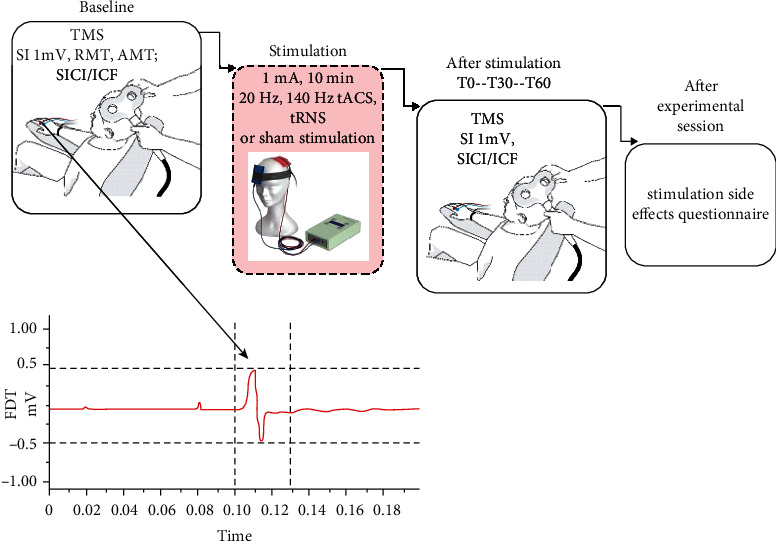
Design of the study. The figure illustrates the procedure for each experimental session. In the beginning of each session, 20 baseline single-pulse MEPs of SI 1 mV amplitude, RMT, AMT, and SICI/ICF were recorded. Afterwards, 1 mA tRNS, 20 Hz tACS, 140 Hz tACS, or sham stimulation was applied over the left primary motor cortex for 10 minutes, and then the SI 1 mV, SICI/ICF were recorded again directly as well as 30 and 60 minutes after stimulation (T0-T60). After finishing each experimental session, the participant was asked to complete a stimulation side effects questionnaire.

For study design see Figure 1. A randomized sham-controlled study with a double-blind, within-subject design was implemented conducting all stimulation conditions in each participant. The order of the stimulation conditions (sham, tRNS, 140 Hz tACS, 20 Hz tACS) was counterbalanced across subjects. Sessions were separated by at least 7 days to avoid carry over effects. In each subject, the experimental sessions were performed at the same time during the day.

The subjects were seated in a comfortable chair with head and arm rests. First, the hotspot (the coil position that produced the largest MEPs of the right FDI) was identified by TMS. Then, the stimulation intensity was adjusted to elicit single-pulse MEPs with peak-to-peak amplitudes of an average of 1 mV and 20 MEPs were recorded prior to stimulation. After determination of SI 1 mV, RMT and AMT were obtained. After measuring AMT, a 15 minutes break followed to avoid an effect of muscle contraction on the next measurements. After this break, SICI/ICF were measured.

Afterwards, 1 mA stimulation (sham, tRNS, 140 Hz tACS, 20 Hz tACS) was administered over 10 minutes. Following stimulation, 20 single test-pulse MEPs, followed by SICI and ICF in counterbalanced order were recorded at intervals of directly after (T0), 30 min (T30) and 60 min (T60) post stimulation. For SI 1 mV, TMS intensity was kept constant for the poststimulation assessment; for the SICI/ICF, TMS intensity was readjusted to obtain single test pulse amplitudes of 1 mV, if needed.

After finishing each experimental session, the participant was asked to complete a stimulation side effects questionnaire adapted from [36]. The questionnaire contains items pertaining to the presence and severity of headaches, change or difficulties in concentration, mood, visual perception, presence of fatigue, and discomforting sensations like pain, tingling, itching, or burning.

Subjects as well as the investigator, who made the MEP measurements, were blinded for stimulation conditions in all studies. The stimulations were done by another investigator.

### 2.5. Data Analysis and Statistics

#### 2.5.1. MEP Analysis

Data analysis was completed manually by visual inspection of offline EMG data. Traces showing any muscle activity prior to the stimulus were removed from the analysis as well as MEPs with a distance of two standard deviations or more to the individual mean.

The MEP means of the participants for the SI 1 mV and the means for each interstimulus interval in SICI (2 ms) and ICF (12 ms) were calculated for the adults and children group before and after stimulation. Poststimulation means of the SI 1 mV threshold were standardized to the prestimulation mean, whereas the mean of the paired stimulation protocols (SICI and ICF) were normalized to the respective single-pulse test condition.

#### 2.5.2. Statistical Comparisons

All statistical analyses were conducted using the computing environment R (version 3.6.1, R Core Team (2016) R: A Language and Environment for Statistical Computing. R Foundation for Statistical Computing, Vienna, Austria. URL https://www.R-project.org/). Throughout all analyses, results were regarded as statistically significant with a two-tailed *p* value of less than 0.05.

Stimulus intensities, baseline MEPs, RMT, and AMT were compared between age groups (children/adolescents, adults) using Wilcoxon signed-rank tests for matched samples, because of failed normal distribution. Furthermore, these baseline values were compared within the children/adolescents and the adults group using the Friedman rank sum test, to exclude baseline differences between the different stimulation conditions.

MEPs for single-pulse (SI 1 mV) and paired-pulse TMS (SICI and ICF) were analyzed separately using linear mixed-effects models. Homogeneity of variances was inspected using Levene's test. SI 1 mV, SICI, and ICF MEPs were log transformed to achieve normal distribution. In all models, we included the maximum number of random effects that allowed the model to converge. Each model included the fixed factors stimulation (sham, tRNS, 140 Hz tACS, 20 Hz tACS), time (T0, T30, T60), age group (children/adolescents, adults), and all corresponding interactions, as well as a random intercept for each participant as random factor.

Differences between baseline and poststimulation (T0, T30, T60) SI 1 mV in each age group were investigated using paired-samples *t*-tests or, in case of failed normal distribution, Wilcoxon signed-rank tests for matched samples with Bonferroni-Holm correction.

In addition, according to our analyses described in Kortuem et al. (2019) [22], the effect of corticospinal activity during sham stimulation on the individual response to tRNS and tACS was investigated. Therefore, response to sham was taken into consideration according to the previously published procedure. Response to sham was evaluated for each individual based on change in MEP amplitudes directly after stimulation (T0) compared to baseline MEP with a Wilcoxon signed-rank test for matched samples. Based on the result of this test, subjects were categorized as either “responder” or “nonresponder” to sham stimulation. Age and baseline parameters of TMS were compared between and within these groups using Wilcoxon signed-rank tests and Friedman tests as described above. To assess whether responders and nonresponders to sham were affected differently by tACS or tRNS, linear mixed models on log-transformed MEPs for single-pulse (SI 1 mV) and paired-pulse TMS (SICI and ICF) were computed for both subgroups. The models contained the fixed factors stimulation (sham, tRNS, 140 Hz tACS, 20 Hz tACS) and time (T0, T30, T60) as well as a random intercept for each participant as random factor. Because of our restricted sample size (see Table 1), we did not include age as an additional factor. Also, differences between baseline and poststimulation (T0, T30, T60) SI 1 mV were investigated for both subgroups using paired-samples *t*-tests or, in case of failed normal distribution, Wilcoxon signed-rank tests for matched samples with Bonferroni-Holm correction.

For all models, degrees of freedom were approximated using the Kenward-Rogers method, analogous to repeated measures ANOVAs [37]. In case of significant *F* values, posthoc tests comparing verum and sham stimulation were performed using the Tukey method.

#### 2.5.3. Adverse Events Questionnaire

The incidence of each side effect was coded in a binary system (yes = 1, no = 0). The severity of each side effect was rated on a numerical analogue scale (NAS) from one to five, one being very mild and five being an extremely high intensity of any given side-effect. Incidence and intensity of adverse effects were compared between age groups using Mann–Whitney *U* test. Furthermore, the number of adverse effects were compared between stimulation conditions using Cochran's *Q* test and McNemar's test; intensity was compared using the Friedman test and Wilcoxon signed-rank test.

## 3. Results

### 3.1. Analyses on Age Groups

There was a significant difference in the MSO % needed to elicit a 1 mV peak-to-peak MEP amplitude for children/adolescents compared to adults, with higher intensities for children/adolescents than for adults (*z* = 3.98, *p* < 0.001). Furthermore, children/adolescents had significantly higher RMT and AMT compared to adults (RMT: *z* = 5.85, *p* < 0.001; AMT: *z* = 4.29, *p* < 0.001). Baseline MEPs did not differ between both groups (*z* = −0.31, *p* = 0.763). Within the groups, these baseline TMS values (stimulus intensities, baseline MEPs, RMT, and AMT) did not differ between the different stimulation conditions. For details, see Table 1.

#### 3.1.1. Single-Pulse MEPs (SI 1 mV)

The linear mixed model for the SI 1 mV MEP amplitudes revealed a significant main effect of stimulation (*p* < 0.001) and a trend for time (*p* = 0.073; see Table 2).

**Figure 2:**
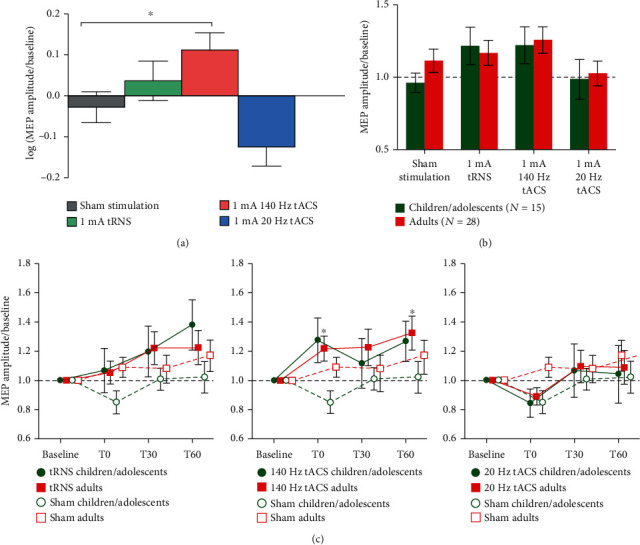
Analyses on age groups. The main effect of age group as well as all interactions were nonsignificant (all *p* > .05). An asterisk indicates *p* < 0.05. (a) Mean SI 1 mV MEP amplitudes (± SEM) of stimulation conditions. The *y*-axis depicts normalized and log-transformed MEP amplitudes. The linear mixed model for the SI 1 mV MEP amplitudes revealed a significant main effect of stimulation (*p* < 0.001). The post hoc tests for all 43 participants showed significant higher amplitudes following 140 Hz stimulation compared to sham stimulation. (b) Mean SI 1 mV MEP amplitudes (± SEM) of stimulation conditions in the children/adolescents and adults group. The *y*-axis depicts normalized MEP amplitudes. The SI 1 mV MEP amplitudes did not differ between age groups for any of the stimulation conditions (all *p* > 0.05). (c) Time course of mean SI 1 mV MEP amplitudes (± SEM) for each age group and each stimulation. The *x*-axis depicts the timepoints before and after stimulation. The *y*-axis displays normalized MEP amplitudes.

The main effect of age group as well as all interactions were nonsignificant (all *p* > 0.05). Our post hoc tests for the factor stimulation showed significant higher SI 1 mV MEP amplitudes following 140 Hz stimulation compared to sham stimulation (*t* (451) = −2.64, *p* = 0.025, see Figure 2(a)). The SI 1 mV MEP amplitudes did not differ between age groups following stimulation for any of the stimulation conditions (all *p* > 0.05; see Figure 2(b)).

Our investigations on differences between baseline and poststimulation SI 1 mV amplitudes showed significant results only for the adult group. Here, amplitudes were significantly increased 0 minutes (*t* (27) = 2.47, *p* = 0.039) and 60 minutes (*t* (27) = 2.68, *p* = 0.037) compared to baseline values following 140 Hz tACS.

#### 3.1.2. Intracortical Inhibition and Facilitation

The mixed models for SICI and ICF MEP amplitudes showed no significant effect of stimulation, time, age group, or any interaction (all *p* > 0.05).

### 3.2. Side-Effects and Sensations

Table 3 displays the frequency and intensity of side effects for children/adolescents and adults. Incidence and intensity did not differ between age groups for any side effect. Comparisons between stimulation conditions were significant only for the adverse effects flickering, tingling, and unpleasantness. Pairwise comparisons to sham stimulation showed increased incidence (*p* < 0.001) and intensity (*z* = −3.71, *p* < 0.001) of flickering for 20 Hz tACS and decreased incidence of flickering for tRNS (*p* = 0.016). Incidence but not intensity of unpleasantness was increased for 20 Hz tACS compared sham stimulation (*p* = 0.031).

### 3.3. Analyses on Response to Sham Groups

**Figure 3:**
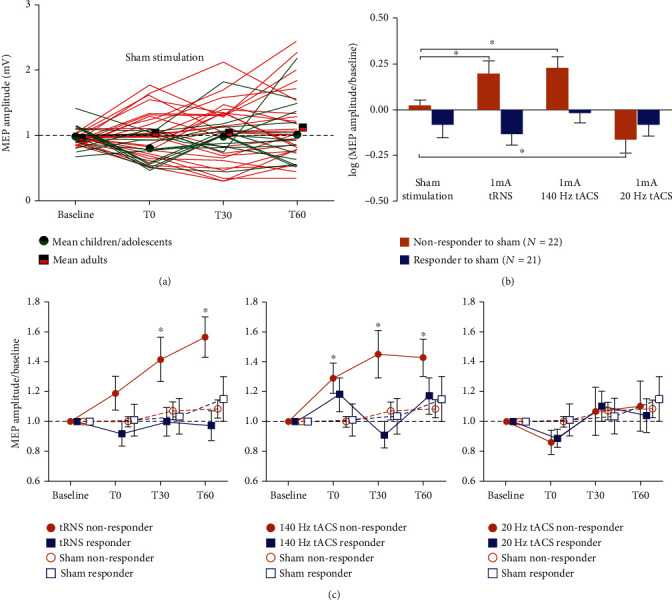
Analyses on response to sham groups: For nonresponders to sham, the linear mixed model for the SI 1 mV MEP amplitudes revealed a significant effect of stimulation and time. An asterisk indicates *p* < 0.05. (a) The individual response to sham for all 43 participants and mean SI 1 mV MEPs for the children/adolescents and adults group. (b) Mean SI 1 mV MEP amplitudes (± SEM) of stimulation conditions. The *y*-axis depicts normalized and log-transformed MEP amplitudes. For nonresponders, post hoc tests showed significant higher SI 1 mV amplitudes following 140 Hz tACS compared to sham stimulation. Also, we found significant lower amplitudes following 20 Hz tACS compared to sham stimulation. (c) Time course of mean SI 1 mV MEP amplitudes (± SEM) for each response group and each stimulation. The *x*-axis depicts the timepoints before and after stimulation. The *y*-axis displays normalized MEP amplitudes. We found increase amplitudes 0, 30, and 60 minutes following 140 Hz tACS, as well as 30 and 60 minutes following tRNS. The inhibitory effect of 20 Hz tACS has only been demonstrated in comparison to sham, but not for the baseline poststimulation comparison.

Figure 3(a) shows the individual response to sham for all 43 participants and mean SI 1 mV MEPs for the children/adolescents and adults group. Our analyses classified 22 participants as nonresponders to sham and 21 participants as responders to sham. Age and baseline parameters of TMS did not differ between nonresponders and responders to sham and within each group for the different stimulation conditions (all *p* > 0.05). For details see Table 1.

#### 3.3.1. Single-Pulse MEPs (SI 1 mV) for Nonresponders to Sham Stimulation

For nonresponders to sham, our linear mixed model for the SI 1 mV MEP amplitudes revealed a significant effect of stimulation (*p* < 0.001) and time (*p* = 0.044; see Table 2). Post hoc tests investigating the stimulation factor showed significant higher SI 1 mV amplitudes following 140 Hz tACS compared to sham stimulation (*t* (231) = −2.91, *p* = 0.012). Also, we found higher amplitudes following tRNS compared to sham stimulation (*t* (231) = −2.447, *p* = 0.016). Additionally, amplitudes were significantly lower following 20 Hz tACS compared to sham stimulation (*t* (231) = 2.66, *p* = 0.016, see Figure 3(b)).

We also conducted post hoc tests investigating the main effect of the factor time. These tests revealed that SI 1 mV amplitudes were generally lower at 0 minutes poststimulation compared to 60 minutes poststimulation (*t* (231) = −2.51, *p* = 0.034).

Our pairwise comparisons on baseline to poststimulation changes in SI 1 mV amplitudes showed increased amplitudes 0 (*z* = 2.46, *p* = 0.018), 30 (*z* = 2.59, *p* = 0.018) and 60 (*z* = 2.98, *p* = 0.008) minutes following 140 Hz tACS. Amplitudes were also increased 30 (*z* = 2.66, *p* = 0.015) and 60 (*z* = 3.27, *p* = 0.003) minutes following tRNS (see Figure 3(c)).

#### 3.3.2. Intracortical Inhibition and Facilitation for Nonresponders to Sham Stimulation

Our investigations on SICI and ICF did not show any significant effect for the stimulation or time factor or for the stimulation × time interaction for nonresponders to sham (all *p* > 0.05).

#### 3.3.3. Single-Pulse MEPs (SI 1 mV) for Responders to Sham Stimulation

Our mixed model for responders to sham investigating the SI 1 mV amplitude did not reveal a significant main effect for stimulation, time, or for the stimulation × time interaction (all *p* > 0.05; see Table 2). The pairwise comparisons on baseline to poststimulation changes in SI 1 mV amplitudes revealed no significant results.

#### 3.3.4. Intracortical Inhibition and Facilitation for Responders to Sham

Our mixed models for SICI and ICF MEP amplitudes did not show any significant effects or interactions for responders to sham (all *p* > 0.05).

## 4. Discussion

The current study aimed at investigating age as well as response to sham stimulation as factors potentially determining the efficacy of tACS and tRNS on motor cortex excitability. We found an effect of verum stimulation that was not influenced by age but by response to sham. Specifically, we observed an excitatory effect of 140 Hz tACS for all participants compared to sham, while tRNS and 20 Hz tACS did not influence corticospinal excitability; yet, these effects did not differ between children/adolescents and adults. Importantly, all types of stimulation were well tolerated by children/adolescents and adults with only minor side effects.

The exploratory analysis of response to sham as a predictor of reponse to verum stimulation showed that only in nonresponders to sham, 140 Hz tACS and tRNS increase and 20 Hz tACS decreases excitability, while responders to sham showed no effect to verum stimulation. For both factors, the effects were limited to single-pulse TMS. For SICI and ICF no effects were observed.

### 4.1. Effect of Age

To the best of our knowledge, no previous study has explored tACS and tRNS effects on motor cortex excitability in a pediatric population. Existing knowledge from adult populations cannot simply be transferred to children, as the child's brain is still developing. For tDCS, it has been demonstrated that the age of subjects and the developmental stage of the brain may affect the efficacy of stimulation and even inverse stimulation effects [24]. In our study, we did not observe differences in stimulation effects between children/adolescents and adults for any of the stimulation conditions. This may have several reasons discussed below.

As expected, 140 Hz tACS led to increased motor cortex excitability with no difference between age groups. This effect is due to the excitatory nature of 1 mA 140 Hz tACS. For anodal tDCS, it has already been shown that excitatory effects occur in the same way in children and adults [24]. The current results are also in line with previous studies showing excitatory effects on motor cortex excitability for frequencies ≥140 Hz [38]. This effect might be based on short-term synaptic plasticity induced by stimulation [19, 39]. Moliadze et al. (2010) reported increased MEP up to one hour following 1 mA 140 Hz tACS and decreased (SICI), an electrophysiological marker of GABA_A_ receptor-mediated inhibition [18]. Beneficial effects of 140 Hz tACS have also been demonstrated for memory consolidation [17].

Similarly, our hypothesis that children/adolescents react to the full spectrum tRNS due to increased neuronal plasticity has been confirmed by our results. Previous studies in adults report increased excitability following high-frequency tRNS [7, 38], showing that the excitatory effect of tRNS derives from higher frequencies (<100 Hz). Different mechanisms have been discussed as explanation for this effect, including stochastic resonance [40] or repetitive opening of Na+ channels [7, 41]. Our results suggest that in both adults and children/adolescents the full spectrum tRNS is not able to influence excitability. However, it is possible that the sample size is simply too small to detect a small-moderate effect of tRNS. The lack of an effect of tRNS in the children/adolescents groups could be due to the comparatively high age of the participants (10-16 years), as an improvement in performance and learning by full-spectrum tRNS was demonstrated for younger children (8.5-10.9 years) [14]. At the same time, no effect of high-frequency tRNS on phoneme processing was observed in children aged 10-16 years [42]. Therefore, based on our current results and those in adults, future studies could investigate whether high-frequency tRNS can lead to excitatory effects in children.

Since beta activity in motor cortical areas is associated with suppression of prepared movements in go-nogo tasks [43, 44], it can be assumed that tACS at 20 Hz would enhance automatic inhibition and therefore decrease motor cortex excitability. However, previous studies report heterogeneous results for 20 Hz tACS applied over the motor cortex area. Our results are in line with several other studies that did not find stimulation effects on motor cortex excitability [45, 46], while Cappon et al. (2016) demonstrated inhibitory effects following 20 Hz stimulation [47]. Unlike Cappon (2016), we did not use a task during stimulation; this activation during stimulation might influence the effects of tACS, since previous studies showed state-dependent effects of tACS [48, 49]. Importantly, no difference between children/adolescents and adults was observed in our study. Additional factors may influence the effect of 20 Hz tACS, such as intensity, phase and duration of stimulation, electrode montage, and activation during stimulation. For example, a recent meta-analysis on the effects of beta tACS showed that only intensities above 1 mA are able to introduce excitatory effects on MCE [25].

Our study is in accordance with earlier studies which show that motor thresholds are higher in children and adolescents compared to adults [24, 50, 51]. Corticospinal tract maturation is a complex process affected by dynamic factors such as synaptic pruning and development, myelination, changes in axonal diameter and length, and organization of pyramidal neuron firing patterns [52–56]. All these factors may also play a role in MEP threshold development. The maturation of the representation of the FDI in the dominant motor cortex is not complete at puberty [57].

### 4.2. Safety and Tolerability

Extreme diligence is required while carrying out NTBS studies in children, as there are few publications, and in case of tACS even no prior experience, concerning the (side) effects of stimulation in this age group. Therefore, the present study is not only relevant with regard to the effects of stimulation on MCE but also concerning the safety and tolerability of stimulation. For tDCS, it has already been shown that this technique is well tolerated by children [58]. Studies conducted in minors did not report considerable side effects, except itching and tingling skin sensations and transient redness of the skin under the electrodes [59–62]. Nevertheless, it should be noted that the use of NTBS in children is a relatively young field of research. One of our previous studies has shown that especially in children and adolescents a more thorough screening for a possible epileptic pathology is necessary [63].

The present study indicates that tACS and tRNS are well tolerated in children and adolescents. The reported side effects were of low to moderate intensity. In addition, there were no differences in the frequency and intensity of reported adverse events between children and adults.

### 4.3. Effect of Response to Sham Stimulation

Our results show different effects depending on the individual response to sham stimulation. Nonresponders to sham showed excitatory effects after 140 Hz and tRNS, as well as inhibitory effects after 20 Hz stimulation. Still, it should be emphasized that the inhibitory effect of 20 Hz tACS has only been demonstrated in comparison to sham, but not for the baseline to poststimulation comparison. The effect has thus not been fully confirmed for all frequencies of stimulation. In contrast, responders to sham showed no stimulation effects. Recently, we demonstrated the influence of response to sham in an adult subgroup for tRNS and 140 Hz stimulation and discussed several possible explanations [22]. Though we were not able to investigate the influence of response to sham in adults versus children/adolescents due to our small sample size for children/adolescents, the effects are robust in the extended sample of adults and children/adolescents combined.

The dependency of stimulation effects on the response to sham might be based on metaplasticity. Neurons are known to be able to change their activity via synaptic plasticity in the form of long-term potentiation (LTP) or depression (LTD) [64]. The basic idea of metaplasticity is that the threshold for activity-dependent synaptic plasticity is not static but dynamic, and it is also a function of the integrated prior activity of the postsynaptic neuron. It refers to synaptic or cellular activity that primes the ability to induce subsequent synaptic plasticity, such as LTP or LTD (for review, see [65, 66]).

However, in our study, we did not measure task-evoked state dependency but rather physiological state dependency. Some current papers and reviews refer to this as “baseline activity;” interchangeably, it is also called “individual physiological brain state” (for a review, see [67]). Therefore, individual physiological state dependency might have contributed to the different outcomes in responders and nonresponders to sham [67, 68]. Following this idea, the interindividual state of neural activity influences the outcome of stimulation [69].

As we discussed previously [22], our results could further support the Bienenstock–Cooper–Munro (BCM) theory [70], which claims that high levels of prior activity favor the induction of LTD, while low levels of prior activity favor LTP [65, 71–73]. However, it should be noted that based on the design of our study the stability of a physiological state as assumed for the BCM theory may not be applicable to our results, given that sham stimulation was only performed once with a gap of least a 7-days to verum stimulation. The BCM theory also does not explain why SICI and ICF were unaffected in our study. If prior neural activity affected LTP/LTD, it would stand to reason that either SICI or ICF measures would be similarly altered (e.g., [74]).

The relationship between SICI and CS intensity is nonlinear and varies between individuals [31, 75]; therefore, using a single CS intensity may contribute to the variability of the outcome. Later studies with threshold-tracking, parameters obtained from the SICI recruitment curve showed better reproducibility [76].

In our study, TMS was delivered at 0.25 Hz. It could be suggested that the TMS with low frequencies may be needed to prevent neuromodulatory effects [77]. For example, Manganotti et al. (2012) shown that single, paired, and transcallosal TMS applied on the sensorimotor areas induced rapid desynchronization over the frontal and central-parietal electrodes mainly in the alpha and beta bands [78]. However, given the low number of stimuli used in the present study, this is unlikely to alter cortical excitability and result in inhibitory effects.

## 5. Limitations

This study is characterized by some limitations. Due to the small sample size, results for tRNS in our study may be vulnerable to overlooking effects (type II statistical error). Therefore, specifically for these null-results, additional studies with larger sample sizes are needed.

Another limiting factor of our study is that we were not able to investigate the effect of response to sham in adults versus children/adolescents. This was due to the comparatively small sample of children and adolescents, which did not allow us to perform a comparison within that group. Therefore, it remains unclear whether there is an interaction between age and response to sham.

In addition, our study investigated only one stimulation intensity. It is conceivable that the stimulation effects vary depending on the intensity and that these intensity effects may also interact with the age of the subjects, as it has been shown for tDCS [24, 79]. However, the aim of the present study was rather to investigate different stimulation conditions and frequencies to lay the foundation for future tACS and tRNS studies in children, especially with regard to safety and tolerability.

Furthermore, the use of neuro-navigation would have been beneficial in order to objectively monitor the coil position and reduce possible distortions caused by the examiner.

## 6. Conclusion

The present study is intended to serve as a basis for further studies investigating tACS and tRNS in the pediatric population as there are currently only few studies. Our results suggest that tACS and tRNS are well-tolerated children and adolescents and no serious adverse events occurred; the evaluation of safety will require longer-lasting investigations. While 140 Hz tACS facilitated excitability, full-spectrum tRNS and 20 Hz tACS did not influence MCE. Interestingly, the effects of stimulation were not modulated by age. At the same time, our study suggests, that the net corticospinal excitability modulation induced during tRNS and 140 Hz and 20 Hz tACS critically depends on the individual sensitivity to sham stimulation. Therefore, it is worth considering the individual response to sham stimulation as a marker for the physiological brain state and an opportunity to understand the variability in the interindividual response to verum stimulation.

## Figures and Tables

**Table 1:** Subject characteristics and threshold before stimulation for age groups (children/adolescents, adults) and response to sham groups (responder to sham, nonresponder to sham). Data are presented in means ± SD.

Age groups	*n*	Sex	Age ± SD	SI_1mV_ (%) ± SD	RMT (%) ± SD	AMT (%) ± SD	Baseline MEP (mV) ± SD
Children/adolescents	15	8M/7F	13.3 ± 2.1				
Sham				61.7 ± 10.5	52.8 ± 4.9	44.8 ± 8.7	0.98 ± 0.18
tRNS				64.1 ± 10.6	54.7 ± 6.5	46.2 ± 8.4	0.94 ± 0.19
140 Hz tACS				62.9 ± 10.6	52.8 ± 6.6	45.1 ± 8.2	0.93 ± 0.16
20 Hz tACS				63.2 ± 10.8	54.5 ± 6.1	46.6 ± 8.9	0.99 ± 0.18
Adults	28	19M/9F	24.4 ± 2.5				
Sham				56.2 ± 10.1	48.5 ± 8.2	40.8 ± 8.1	0.96 ± 0.08
tRNS				56.7 ± 10.8	48.8 ± 9.1	40.9 ± 8.7	0.96 ± 0.09
140 Hz tACS				55.5 ± 10.3	47.4 ± 8.6	39.1 ± 8.1	0.98 ± 0.09
20 Hz tACS				55.5 ± 10.8	47.9 ± 8.9	40.2 ± 7.6	0.98 ± 0.09
Response to sham groups	*n*	Sex	Age ± SD (adults/children)	SI_1mV_ (%) ± SD	RMT (%) ± SD	AMT (%) ± SD	Baseline MEP (mV) ± SD
Responder to sham	21	13M/8F	20.9 ± 4.7 (16/5)				
Sham				58.8 ± 8.5	50.4 ± 6.8	43.1 ± 7.4	0.97 ± 0.11
tRNS				58.3 ± 10.5	49.3 ± 8.1	42.8 ± 8.5	0.94 ± 0.14
140 Hz tACS				57.3 ± 8.7	48.1 ± 6.3	41.1 ± 6.7	0.98 ± 0.09
20 Hz tACS				57.9 ± 7.9	49.1 ± 6.5	42.3 ± 6.6	0.94 ± 0.11
Nonresponder to sham	22	14M/8F	20.1 ± 6.9 (12/10)				
Sham				57.5 ± 12.2	48.4 ± 8.7	41.3 ± 9.4	0.96 ± 0.14
tRNS				60.2 ± 12.1	51.3 ± 9.7	42.7 ± 9.5	0.97 ± 0.11
140 Hz tACS				58.9 ± 12.8	49.3 ± 10.6	41.3 ± 10.2	0.96 ± 0.15
20 Hz tACS				58.4 ± 14.1	49.8 ± 10.9	42.5 ± 10.3	1.03 ± 0.14

SD: standard deviation; F: female; M: male; SI: stimulus intensity; RMT: resting motor threshold; AMT: active motor threshold; MEP: motor evoked potential.

**Table 2:** The results of linear mixed models (LMM) performed on SI 1 mV, SICI, and ICF for all participants, nonresponder and responder to sham.

	Numerator *df*	Denominator *df*	*F* value	*p* value
SI 1 mV				
Stimulation	3	451	6.69	**<0.001**
Time	2	451	2.62	0.073
Age group	1	41	0.57	0.451
Stimulation × age group	3	451	0.31	0.816
Time × age group	2	451	0.34	0.705
Stimulation × time	6	451	1.01	0.423
Stimulation × time × age group	6	451	0.59	0.736
SICI				
Stimulation	3	448	0.31	0.816
Time	2	448	0.11	0.897
Age group	1	41	1.84	0.182
Stimulation × age group	3	448	0.82	0.486
Time × age group	2	448	1.19	0.304
Stimulation × time	6	448	0.36	0.901
Stimulation × time × age group	6	448	1.63	0.135
ICF				
Stimulation	3	448	0.19	0.901
Time	2	448	0.85	0.425
Age group	1	41	0.64	0.426
Stimulation × age group	3	448	0.28	0.839
Time × age group	2	448	0.67	0.509
Stimulation × time	6	448	0.28	0.839
Stimulation × time × age group	6	448	1.15	0.328
Nonresponder to sham	Numerator *df*	Denominator *df*	*F* value	*p* value
SI 1 mv				
Stimulation	3	231	13.14	**< 0.001**
Time	2	231	3.16	**0.044**
Stimulation × time	6	231	0.51	0.803
SICI				
Stimulation	3	228	0.71	0.546
Time	2	228	1.91	0.149
Stimulation × time	6	228	0.67	0.672
ICF				
Stimulation	3	228	1.29	0.277
Time	2	228	0.31	0.729
Stimulation × time	6	228	0.85	0.531
*Responder to sham*	Numerator *df*	Denominator *df*	*F* value	*p* value
*SI 1 mv*				
Stimulation	3	231	0.76	0.513
Time	2	231	0.27	0.764
Stimulation × time	6	231	1.02	0.411
SICI				
Stimulation	3	220	0.95	0.414
Time	2	220	1.48	0.229
Stimulation × time	6	220	0.09	0.997
ICF				
Stimulation	3	220	0.12	0.942
Time	2	220	0.51	0.601
Stimulation × time	6	220	1.17	0.319

**Table 3:** Side-effects and sensations. Incidence (sum) and intensity (scale 1-4, Ø) of side effects for the different stimulation conditions in the children/adolescents and adult subgroups.

	Incidence				Intensity			
	Sham	tRNS	140 Hz	20 Hz	Sham	tRNS	140 Hz	20 Hz
Children/adolescents								
Burning	1	0	0	0	2	—	—	—
Difficulties in concentration	1	1	1	1	3	—	3	1
Fatigue	2	0	4	2	2.5	1	2.5	1
Flickering	2	2	0	6	1.5	—	—	2.5
Headache	0	1	0	0	—	1	—	—
Itching	1	0	1	2	1	1	1	1.5
Nervousness	0	0	0	1	—	—	—	1
Pain	1	2	0	1	2	—	—	2
Tingling	1	0	1	3	1	1	1	1.7
Unpleasantness	1	2	0	3	2	—	—	1
Adults								
Burning	2	1	2	2	1.5	1	1	1.5
Difficulties in concentration	0	2	0	1	—	1.5	—	2
Fatigue	7	10	7	6	2.4	1.5	1.6	1.8
Flickering	6	2	1	16	1.5	2	2	2.1
Headache	1	1	0	0	1	1	—	—
Itching	1	0	3	4	2	—	1.7	1.3
Nervousness	1	2	1	3	2	1.5	1	1
Pain	2	3	0	3	1.5	1	—	1.7
Tingling	5	1	2	8	1.2	2	2	1.5
Unpleasantness	1	2	1	5	1	1.5	1	1.2

## Data Availability

Due to ethical restriction, the data from this study will not be able to be accessible from public domain. The data are available from the corresponding authors upon request. Maike Splittgerber and Vera Moliadze, Institute of Medical Psychology and Medical Sociology, University Medical Center Schleswig Holstein, Kiel University, Kiel, Germany (splittgerber@med-psych.uni-kiel.de, moliadze@med-psych.uni-kiel.de).

## References

[1] Huang Y. Z., Lu M. K., Antal A. (2017). Plasticity induced by non-invasive transcranial brain stimulation: a position paper. *Clinical Neurophysiology*.

[2] Herrmann C. S., Rach S., Neuling T., Strüber D. (2013). Transcranial alternating current stimulation: a review of the underlying mechanisms and modulation of cognitive processes. *Frontiers in Human Neuroscience*.

[3] Antal A., Paulus W. (2013). Transcranial alternating current stimulation (tACS). *Frontiers in Human Neuroscience*.

[4] Paulus W. (2011). Transcranial electrical stimulation (tES - tDCS; tRNS, tACS) methods. *Neuropsychological Rehabilitation*.

[5] Thut G., Schyns P. G., Gross J. (2011). Entrainment of perceptually relevant brain oscillations by non-invasive rhythmic stimulation of the human brain. *Frontiers in Psychology*.

[6] Pavan A., Ghin F., Contillo A., Milesi C., Campana G., Mather G. (2019). Modulatory mechanisms underlying high-frequency transcranial random noise stimulation (hf-tRNS): a combined stochastic resonance and equivalent noise approach. *Brain Stimulation*.

[7] Terney D., Chaieb L., Moliadze V., Antal A., Paulus W. (2008). Increasing human brain excitability by transcranial high-frequency random noise stimulation. *The Journal of Neuroscience*.

[8] Antal A., Herrmann C. S. (2016). Transcranial alternating current and random noise stimulation: possible mechanisms. *Neural Plasticity*.

[9] Finisguerra A., Borgatti R., Urgesi C. (2019). Non-invasive brain stimulation for the rehabilitation of children and adolescents with neurodevelopmental disorders: a systematic review. *Frontiers in Psychology*.

[10] Brunoni A. R., Nitsche M. A., Bolognini N. (2012). Clinical research with transcranial direct current stimulation (tDCS): challenges and future directions. *Brain Stimulation*.

[11] Davis N. J. (2014). Transcranial stimulation of the developing brain: a plea for extreme caution. *Frontiers in Human Neuroscience*.

[12] Maslen H., Earp B. D., Cohen Kadosh R., Savulescu J. (2014). Brain stimulation for treatment and enhancement in children: an ethical analysis. *Frontiers in Human Neuroscience*.

[13] Kadosh R. C., Levy N., O'Shea J., Shea N., Savulescu J. (2012). The neuroethics of non-invasive brain stimulation. *Current Biology*.

[14] Looi C. Y., Lim J., Sella F. (2017). Transcranial random noise stimulation and cognitive training to improve learning and cognition of the atypically developing brain: a pilot study. *Scientific Reports*.

[15] Baker S. N. (2007). Oscillatory interactions between sensorimotor cortex and the periphery. *Current Opinion in Neurobiology*.

[16] Pogosyan A., Gaynor L. D., Eusebio A., Brown P. (2009). Boosting cortical activity at beta-band frequencies slows movement in humans. *Current Biology*.

[17] Ambrus G. G., Pisoni A., Primassin A., Turi Z., Paulus W., Antal A. (2015). Bi-frontal transcranial alternating current stimulation in the ripple range reduced overnight forgetting. *Frontiers in Cellular Neuroscience*.

[18] Moliadze V., Antal A., Paulus W. (2010). Boosting brain excitability by transcranial high frequency stimulation in the ripple range. *The Journal of Physiology*.

[19] Chaieb L., Paulus W., Antal A. (2011). Evaluating aftereffects of short-duration transcranial random noise stimulation on cortical excitability. *Neural Plasticity*.

[20] Fertonani A., Pirulli C., Miniussi C. (2011). Random noise stimulation improves neuroplasticity in perceptual learning. *Journal of Neuroscience*.

[21] Popescu T., Krause B., Terhune D. B. (2016). Transcranial random noise stimulation mitigates increased difficulty in an arithmetic learning task. *Neuropsychologia*.

[22] Kortuem V., Kadish N. E., Siniatchkin M., Moliadze V. (2019). Efficacy of tRNS and 140 Hz tACS on motor cortex excitability seemingly dependent on sensitivity to sham stimulation. *Experimental Brain Research*.

[23] Moliadze V., Atalay D., Antal A., Paulus W. (2012). Close to threshold transcranial electrical stimulation preferentially activates inhibitory networks before switching to excitation with higher intensities. *Brain Stimulation*.

[24] Moliadze V., Schmanke T., Andreas S., Lyzhko E., Freitag C. M., Siniatchkin M. (2015). Stimulation intensities of transcranial direct current stimulation have to be adjusted in children and adolescents. *Clinical Neurophysiology*.

[25] Wischnewski M., Schutter D. J. L. G., Nitsche M. A. (2019). Effects of beta-tACS on corticospinal excitability: a meta-analysis. *Brain Stimulation*.

[26] Oldfield R. C. (1971). The assessment and analysis of handedness: the Edinburgh inventory. *Neuropsychologia*.

[27] Moliadze V., Antal A., Paulus W. (2010). Electrode-distance dependent after-effects of transcranial direct and random noise stimulation with extracephalic reference electrodes. *Clinical Neurophysiology*.

[28] Awiszus F. (2003). Chapter 2 TMS and threshold hunting. *Transcranial Magnetic Stimulation and Transcranial Direct Current Stimulation, Proceedings of the 2nd International Transcranial Magnetic Stimulation (TMS) and Transcranial Direct Current Stimulation (tDCS) Symposium*.

[29] Rothwell J. C., Hallett M., Berardelli A., Eisen A., Rossini P., Paulus W. (1999). Magnetic stimulation: motor evoked potentials. The International Federation of Clinical Neurophysiology. *Electroencephalography and Clinical Neurophysiology. Supplement*.

[30] Kujirai T., Caramia M. D., Rothwell J. C. (1993). Corticocortical inhibition in human motor cortex. *The Journal of Physiology*.

[31] Rossini P. M., Burke D., Chen R. (2015). Non-invasive electrical and magnetic stimulation of the brain, spinal cord, roots and peripheral nerves: basic principles and procedures for routine clinical and research application. An updated report from an I.F.C.N. committee. *Clinical Neurophysiology*.

[32] Di Lazzaro V., Oliviero A., Meglio M. (2000). Direct demonstration of the effect of lorazepam on the excitability of the human motor cortex. *Clinical Neurophysiology*.

[33] Wagle-Shukla A., Ni Z., Gunraj C. A., Bahl N., Chen R. (2009). Effects of short interval intracortical inhibition and intracortical facilitation on short interval intracortical facilitation in human primary motor cortex. *The Journal of Physiology*.

[34] Ziemann U., Rothwell J. C., Ridding M. C. (1996). Interaction between intracortical inhibition and facilitation in human motor cortex. *The Journal of Physiology*.

[35] Fresnoza S., Christova M., Feil T. (2018). The effects of transcranial alternating current stimulation (tACS) at individual alpha peak frequency (iAPF) on motor cortex excitability in young and elderly adults. *Experimental Brain Research*.

[36] Poreisz C., Boros K., Antal A., Paulus W. (2007). Safety aspects of transcranial direct current stimulation concerning healthy subjects and patients. *Brain Research Bulletin*.

[37] Kenward M. G., Roger J. H. (1997). Small sample inference for fixed effects from restricted maximum likelihood. *Biometrics*.

[38] Dissanayaka T., Zoghi M., Farrell M., Egan G. F., Jaberzadeh S. (2017). Does transcranial electrical stimulation enhance corticospinal excitability of the motor cortex in healthy individuals? A systematic review and meta-analysis. *European Journal of Neuroscience*.

[39] Citri A., Malenka R. C. (2008). Synaptic plasticity: multiple forms, functions, and mechanisms. *Neuropsychopharmacology*.

[40] Wiesenfeld K., Moss F. (1995). Stochastic resonance and the benefits of noise: from ice ages to crayfish and SQUIDs. *Nature*.

[41] Schoen I., Fromherz P. (2008). Extracellular stimulation of mammalian neurons through repetitive activation of Na+ channels by weak capacitive currents on a silicon chip. *Journal of Neurophysiology*.

[42] Rufener K. S., Krauel K., Meyer M., Heinze H. J., Zaehle T. (2019). Transcranial electrical stimulation improves phoneme processing in developmental dyslexia. *Brain Stimulation*.

[43] Swann N., Tandon N., Canolty R. (2009). Intracranial EEG reveals a time- and frequency-specific role for the right inferior frontal gyrus and primary motor cortex in stopping initiated responses. *The Journal of Neuroscience*.

[44] Swann N. C., Cai W., Conner C. R. (2012). Roles for the pre-supplementary motor area and the right inferior frontal gyrus in stopping action: electrophysiological responses and functional and structural connectivity. *NeuroImage*.

[45] Rjosk V., Kaminski E., Hoff M. (2016). Transcranial alternating current stimulation at beta frequency: lack of immediate effects on excitation and interhemispheric inhibition of the human motor cortex. *Frontiers in Human Neuroscience*.

[46] Wach C., Krause V., Moliadze V., Paulus W., Schnitzler A., Pollok B. (2013). Effects of 10 Hz and 20 Hz transcranial alternating current stimulation (tACS) on motor functions and motor cortical excitability. *Behavioural Brain Research*.

[47] Cappon D., D'Ostilio K., Garraux G., Rothwell J., Bisiacchi P. (2016). Effects of 10 Hz and 20 Hz transcranial alternating current stimulation on automatic motor control. *Brain Stimulation*.

[48] Feurra M., Pasqualetti P., Bianco G., Santarnecchi E., Rossi A., Rossi S. (2013). State-dependent effects of transcranial oscillatory currents on the motor system: what you think matters. *Journal of Neuroscience*.

[49] Fusca M., Ruhnau P., Neuling T., Weisz N. (2018). Local network-level integration mediates effects of transcranial alternating current stimulation. *Brain Connectivity*.

[50] Frye R. E., Rotenberg A., Ousley M., Pascual-Leone A. (2007). Transcranial magnetic stimulation in child neurology: current and future directions. *Journal of Child Neurology*.

[51] Garvey M. A., Mall V. (2008). Transcranial magnetic stimulation in children. *Clinical Neurophysiology*.

[52] Chiappa K., Cros D., Day B., Fang J., Macdonell R., Mavroudakis N. (1991). Magnetic stimulation of the human motor cortex: ipsilateral and contralateral facilitation effects. *Electroencephalography and Clinical Neurophysiology. Supplement*.

[53] Eyre J. A., Miller S., Ramesh V. (1991). Constancy of central conduction delays during development in man: investigation of motor and somatosensory pathways. *The Journal of Physiology*.

[54] Eyre J. A., Taylor J. P., Villagra F., Smith M., Miller S. (2001). Evidence of activity-dependent withdrawal of corticospinal projections during human development. *Neurology*.

[55] Papadelis C., Kaye H., Shore B., Snyder B., Grant P. E., Rotenberg A. (2019). Maturation of Corticospinal Tracts in Children With Hemiplegic Cerebral Palsy Assessed by Diffusion Tensor Imaging and Transcranial Magnetic Stimulation. *Frontiers in Human Neuroscience*.

[56] Paus T., Collins D. L., Evans A. C., Leonard G., Pike B., Zijdenbos A. (2001). Maturation of white matter in the human brain: a review of magnetic resonance studies. *Brain Research Bulletin*.

[57] Garvey M. A., Ziemann U., Bartko J. J., Denckla M. B., Barker C. A., Wassermann E. M. (2003). Cortical correlates of neuromotor development in healthy children. *Clinical Neurophysiology*.

[58] Krishnan C., Santos L., Peterson M. D., Ehinger M. (2015). Safety of noninvasive brain stimulation in children and adolescents. *Brain Stimulation*.

[59] Andrade A. C., Magnavita G. M., Allegro J. V. B. N. (2013). Feasibility of transcranial direct current stimulation use in children aged 5 to 12 years. *Journal of Child Neurology*.

[60] Antal A., Alekseichuk I., Bikson M. (2017). Low intensity transcranial electric stimulation: safety, ethical, legal regulatory and application guidelines. *Clinical Neurophysiology*.

[61] Mattai A., Miller R., Weisinger B. (2011). Tolerability of transcranial direct current stimulation in childhood-onset schizophrenia. *Brain Stimulation*.

[62] Moliadze V., Andreas S., Lyzhko E. (2015). Ten minutes of 1 mA transcranial direct current stimulation was well tolerated by children and adolescents: Self-reports and resting state EEG analysis. *Brain Research Bulletin*.

[63] Splittgerber M., Japaridze N., Sierawska A. (2020). First generalized tonic clonic seizure in the context of pediatric tDCS – A case report. *Neurophysiologie Clinique*.

[64] Abbott L. F., Nelson S. B. (2000). Synaptic plasticity: taming the beast. *Nature Neuroscience*.

[65] Karabanov A., Ziemann U., Hamada M. (2015). Consensus paper: probing homeostatic plasticity of human cortex with non-invasive transcranial brain stimulation. *Brain Stimulation*.

[66] Muller-Dahlhaus F., Ziemann U. (2015). Metaplasticity in human cortex. *The Neuroscientist*.

[67] Krause B., Kadosh R. C. (2014). Not all brains are created equal: the relevance of individual differences in responsiveness to transcranial electrical stimulation. *Frontiers in Systems Neuroscience*.

[68] Silvanto J., Muggleton N., Walsh V. (2008). State-dependency in brain stimulation studies of perception and cognition. *Trends in Cognitive Sciences*.

[69] Ammann C., Lindquist M. A., Celnik P. A. (2017). Response variability of different anodal transcranial direct current stimulation intensities across multiple sessions. *Brain Stimulation*.

[70] Bienenstock E. L., Cooper L. N., Munro P. W. (1982). Theory for the development of neuron selectivity: orientation specificity and binocular interaction in visual cortex. *The Journal of Neuroscience*.

[71] Lang N., Nitsche M. A., Paulus W., Rothwell J. C., Lemon R. N. (2004). Effects of transcranial direct current stimulation over the human motor cortex on corticospinal and transcallosal excitability. *Experimental Brain Research*.

[72] Siebner H. R., Lang N., Rizzo V. (2004). Preconditioning of low-frequency repetitive transcranial magnetic stimulation with transcranial direct current stimulation: evidence for homeostatic plasticity in the human motor cortex. *The Journal of Neuroscience*.

[73] Ziemann U., Paulus W., Nitsche M. A. (2008). Consensus: motor cortex plasticity protocols. *Brain Stimulation*.

[74] Golaszewski S. M., Bergmann J., Christova M. (2010). Increased motor cortical excitability after whole-hand electrical stimulation: a TMS study. *Clinical Neurophysiology*.

[75] Chen R., Tam A., Bütefisch C. (1998). Intracortical inhibition and facilitation in different representations of the human motor cortex. *Journal of Neurophysiology*.

[76] Samusyte G., Bostock H., Rothwell J., Koltzenburg M. (2018). Short-interval intracortical inhibition: comparison between conventional and threshold-tracking techniques. *Brain Stimulation*.

[77] Chen R., Gerloff C., Classen J., Wassermann E. M., Hallett M., Cohen L. G. (1997). Safety of different inter-train intervals for repetitive transcranial magnetic stimulation and recommendations for safe ranges of stimulation parameters. *Electroencephalography and Clinical Neurophysiology*.

[78] Manganotti P., Formaggio E., Storti S. F. (2012). Time-frequency analysis of short-lasting modulation of EEG induced by intracortical and transcallosal paired TMS over motor areas. *Journal of Neurophysiology*.

[79] Moliadze V., Lyzhko E., Schmanke T., Andreas S., Freitag C. M., Siniatchkin M. (2018). 1 mA cathodal tDCS shows excitatory effects in children and adolescents: Insights from TMS evoked N100 potential. *Brain Research Bulletin*.

